# lncRNAs in Non-Malignant Tissue Have Prognostic Value in Colorectal Cancer

**DOI:** 10.3390/ijms19092672

**Published:** 2018-09-08

**Authors:** Jana-Aletta Thiele, Petr Hosek, Eva Kralovcova, Pavel Ostasov, Vaclav Liska, Jan Bruha, Ondrej Vycital, Jachym Rosendorf, Alena Opattova, Josef Horak, Milena Kralickova, Pavel Vodicka, Pavel Pitule

**Affiliations:** 1 Biomedical Centre, Faculty of Medicine in Pilsen, Charles University, Alej Svobody 76, 32300 Pilsen, Czech Republic; Jana.A.Thiele@lfp.cuni.cz (J.-A.T.); petr.hosek@lfp.cuni.cz (P.H.); Eva.Kralovcova@lfp.cuni.cz (E.K.); Pavel.Ostasov@lfp.cuni.cz (P.O.); liskav@fnplzen.cz (V.L.); bruhaj@fnplzen.cz (J.B.); vycitalo@fnplzen.cz (O.V.); rosendorfj@fnplzen.cz (J.R.); alenaopattova@gmail.com (A.O.); horajozka@seznam.cz (J.H.); Milena.Kralickova@lfp.cuni.cz (M.K.); pvodicka@biomed.cas.cz (P.V.); 2 Department of Surgery, Faculty of Medicine in Pilsen, Charles University, Alej Svobody 80, 32300 Pilsen, Czech Republic; 3 Department of Molecular Biology of Cancer, Institute of Experimental Medicine of the Czech Academy of Sciences, Videnska 1083, 14220 Prague, Czech Republic; 4 Institute of Biology and Medical Genetics, First Faculty of Medicine, Albertov 4, Charles University, 12800 Prague, Czech Republic; 5 Department of Histology and Embryology, Faculty of Medicine in Pilsen, Charles University, Karlovarska 48, 30166 Pilsen, Czech Republic

**Keywords:** colorectal carcinoma, lncRNA, lncRNA ratio, MIR155HG, CCAT1, PCAT1

## Abstract

Although colorectal cancer (CRC) is the third most frequent cause of cancer related death in Europe, clinically relevant biomarkers for therapy guidance and prognosis are insufficiently reliable. Long non-coding RNAs (lncRNAs) are RNAs over 200 nucleotides long that are not translated into proteins but can influence biological processes. There is emerging evidence for their involvement in solid cancer as oncogenes, tumour suppressors or regulators of cell proliferation and metastasis development. The goal of this study was to evaluate the prognostic effect of selected lncRNAs in a retrospective study on CRC patients from the Czech Republic. We used a quantitative PCR approach to measure the expression in paired non-malignant and tumour tissue samples of CRC patients of nine lncRNAs previously shown to be involved in cancer progression—*ANRIL*, *CCAT1*, *GAS5*, *linc-ROR*, *MALAT1*, *MIR155HG*, *PCAT1*, *SPRY4-IT1* and *TUG1*. Associations between expression and expression ratios and clinical characteristics and survival were assessed by using univariable Cox proportional hazards models, Kaplan-Meier estimations with the Gehan-Wilcoxon test, the Mann-Whitney U test, the Kruskal-Wallis test and Spearman’s correlations. A comparison of expression in tumour tissue (TT) and non-malignant mucosa tissue (MT) showed significant upregulation of *CCAT1* and *linc-ROR* in TT (*p* < 0.001 and *p* = 0.001, respectively) and downregulation of *ANRIL*, *MIR155HG* and *MALAT1* (*p* = 0.001, *p* = 0.010, *p* = 0.001, respectively). *Linc-ROR* was significantly associated with the presence of synchronous metastases (*p* = 0.033). For individual tissue types, lower *MIR155HG* expression in TT was correlated with both shorter overall survival (*p* = 0.008) and shorter disease-free survival (*p* = 0.040). In MT, expression ratios of *CCAT1*/*ANRIL* and *CCAT1*/*MIR155HG* were associated with overall survival (*p* = 0.005 and *p* = 0.006, respectively). Our results revealed that changes in expression of lncRNAs between MT and TT hold potential to be used as prognostic biomarkers in CRC patients. Moreover, the ratios of *CCAT1* to *ANRIL* and *MIR155HG* in MT also exhibit potential for prognosis assessment without tumour sampling. Our results also indicate that cancer progression is associated with detrimental system-wide changes in patient tissue, which might govern patient survival even after successful elimination of tumour or cancerous cells.

## 1. Introduction

In 2017, colorectal cancer (CRC) was predicted to be the second and third leading cause of cancer-related death in men and women, respectively, in Europe [1]. Early stages of CRC are usually asymptomatic with a lack of biomarkers for their early detection, less than 40% of patients are therefore diagnosed with localized disease. Early diagnosis has a severe impact on 5 year survival rate, dropping from 90% for localized disease to 12% for patients with distant metastasis [2,3,4]. This threat makes it more pressing to discover new biomarkers for all disease stages and potential therapeutic targets in order to better predict disease evolution and understand the epigenetic regulation of gene expression that promotes metastasis.

Long non-coding RNAs (lncRNAs) have been discovered as regulators of gene expression and are often abnormally expressed in tumour tissue [5,6]. Recent studies have attempted to uncover functionalities of lncRNAs related to their regulation of gene expression, apoptosis, or proliferation as well as their role in the metastatic potential of cancer cells [7,8,9,10,11,12,13]. Based on these results, we chose a set of nine lncRNAs for a study to evaluate their prognostic value on a group of Czech CRC patients. The lncRNAs were selected according their potential to promote or influence the following processes: CRC progression in general (*TUG1*, *GAS5* and *MALAT1*) [10,11,14,15], cell proliferation (*SPRY4-IT1*, *GAS5* and *CCAT-1*) [10,12,16], cell migration (*ANRIL*) [17], apoptosis (*PCAT1*) [8], regulation of tumour suppressors such as p53 (*linc-ROR*) [18] and inhibition of epithelial-mesenchymal transition (EMT) in other cancer types (*MIR155HG*) [19] (references, full names and functions for each selected lncRNA are stated in Table S1). 

So far, the most common approach of oncological studies that have addressed lncRNAs, has been to analyse differential expression between tumour and healthy tissue [8,9,20]. However, in this study we also use additional approaches such as analysis of normalized gene expression levels within individual tissue types or expression ratios of two non-reference genes, that proved their usefulness in several studies [21,22] but have not been applied to lncRNAs to our knowledge. 

The aim of our retrospective study is to correlate expression levels of the selected lncRNAs (*ANRIL*, *linc-ROR*, *CCAT1*, *PCAT1*, *SPRY4-IT1*, *TUG1*, *GAS5*, *MALAT1* and *MIR155HG*) measured by q -PCR with survival and clinicopathological features of CRC patients. To explore the potential of lncRNAs as biomarkers, we analysed their differential expression in tumour tissue (TT) with respect to non-malignant mucosa tissue (MT), their normalized expression in individual tissue types and the expression ratios of pairs of lncRNAs separately in MT and TT. Combination of these methods allowed us to gain unique data that might be used for future clinical studies. 

## 2. Results

### 2.1. Patient´s Characteristics

Clinical summary of CRC patients enrolled in the study is presented in Table 1. The median follow-up time was 4.6 years. In our patient group, we observed a three-year OS (overall survival) of 85.1% and three-year DFS (disease-free survival) of 72.8%. For our patient group the median OS and DFS was not reached.

### 2.2. Expression Fold Change of lncRNAs (long non-coding RNAs) in Tumour Tissue (TT) Compared to Non-Malignant Tissue (MT)

**Figure 1:**
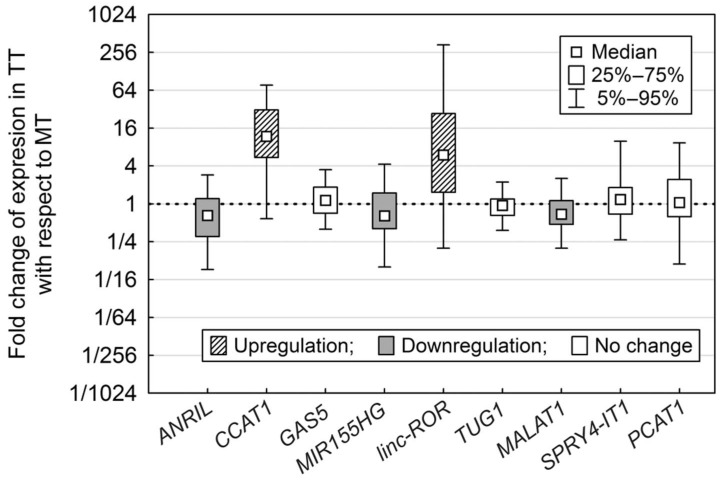
Expression of nine lncRNAs (long non-coding RNAs) in tumour tissue compared to non-malignant tissue. Boxplots of nine lncRNAs showing the fold change of their expression in tumour tissue vs. non-malignant tissue. Significantly up- or downregulated lncRNAs (*p* < 0.05) are marked.

Quantitative PCR analysis showed differences in lncRNA expression between the tested MT and TT samples (Figure 1). Overexpression in TT was observed for the lncRNAs *CCAT1* (*p* < 0.0001) and *linc-ROR* (*p* = 0.0009). Significant downregulation in TT was reported for the lncRNAs *ANRIL* (*p* = 0.0010), *MIR155HG* (*p* = 0.0101) and *MALAT1* (*p* = 0.0006).

When investigating the expression of lncRNAs as a continuous predictor of DFS and OS using a univariable Cox proportional hazards model, significant associations with DFS were observed for *MIR155HG* (*p* = 0.0402) with a log2 hazard rate (HR) of 0.68 (i.e., the risk of disease progression is decreased by 32% each time *MIR155HG* expression fold change is doubled). Similar tendency was observed for *PCAT1* (*p* = 0.0441; HR = 0.73). An association with OS was only observed for *MIR155HG* (*p* = 0.0079; HR = 0.57).

**Figure 2:**
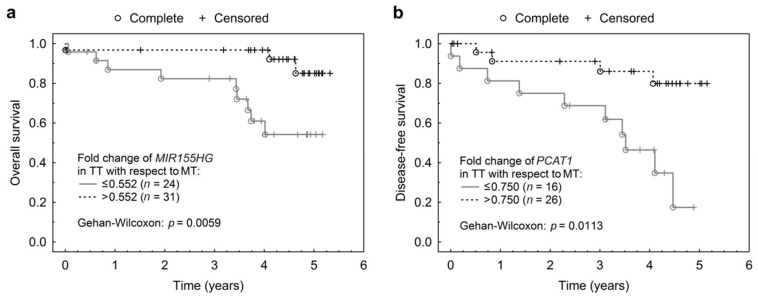
Association between *MIR155HG* and *PCAT1* expression fold change and survival. (**a**) Kaplan Meier curve using optimized thresholds for fold change of *MIR155HG* and its association with OS (overall survival) and (**b**) fold change of *PCAT1* and its association with DFS (disease-free survival).

Subsequent comparison of Kaplan-Meier survival curves (Figure 2) for patient groups based on lncRNA expression using an optimized threshold confirmed the association between *MIR155HG* expression change and OS (*p* = 0.0059, Figure 2a) as well as *PCAT1* expression change and DFS (*p* = 0.0113, Figure 2b).

**Figure 3:**
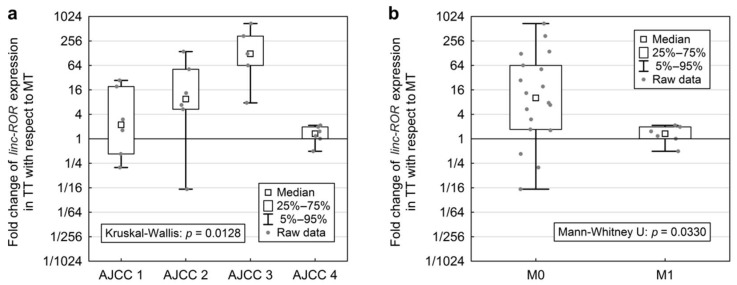

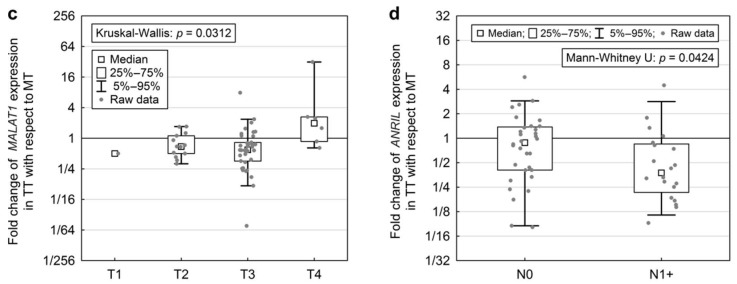
Significant associations between lncRNAs expression fold change in tumour tissue (TT) vs. healthy tissue (MT) and tumour staging. Displayed is the ratio between healthy tissue (MT) and tumour tissue (TT). A value above 1 represents elevated expression in TT. (**a**) The change in expression of *linc-ROR* differed significantly within the categories of AJCC staging and was largest in AJCC stage 3 samples; (**b**) The change in expression of *linc-ROR* was significantly higher in patients without distant metastases; (**c**) For *MALAT1* the fold change varied significantly between the T stages; (**d**) Change in *ANRIL* expression change was significantly higher in patients without lymph node involvement (N0).

When analysing the associations between lncRNA expression changes and clinical characteristics (Figure 3), the fold change of *linc-ROR* in TT compared to MT varied significantly between the four AJCC stages (Kruskal-Wallis: *p* = 0.0128, Figure 3a) and *linc-ROR* fold change was significantly higher in patients without distant metastases (Mann-Whitney U: *p* = 0.0330, Figure 3b). The expression changes of *MALAT1* varied significantly between T stages (Kruskal-Wallis: *p* = 0.0312, Figure 3c) and was significantly different between patients with T3 and T4 (*p* = 0.0244, Multiple rank comparison). *ANRIL* fold change in TT was significantly higher in patients without lymph node involvement (Mann-Whitney U: *p* = 0.0424, Figure 3d). With regards to the other lncRNAs, Spearman correlations showed positive association of age with higher expression of *TUG1* in TT (*p* = 0.0362, Figure S1).

No correlation was detected between lncRNA expression change and gender, cancer grade (G) or tumour location.

### 2.3. Expression of lncRNAs in Non-malignant and Tumour Tissue 

Normalized expression of the nine tested lncRNAs in the two separate tissue types was analysed to test if their relative expression was associated with survival and/or clinical data.

**Figure 4:**
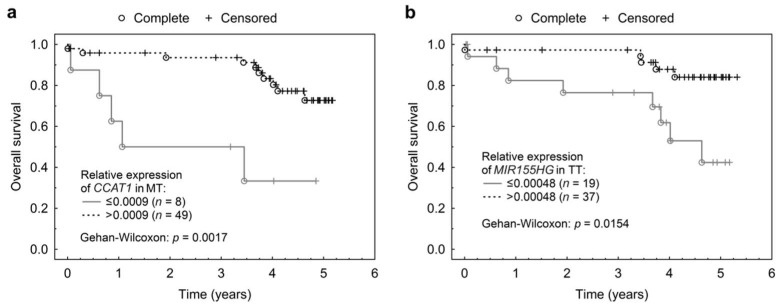
Associations of lncRNA normalized expressions in MT and TT with overall survival. (**a**) Kaplan Meier curve for the relationship between *CCAT1* expression in MT with OS and (**b**) Kaplan Meier curve for *MIR155HG* expression in TT and its association with OS.

Significant associations between lncRNA expressions and OS was detected using a univariable Cox proportional hazard model for *CCAT1* in MT (*p* = 0.0329; HR = 0.63) and *MIR155HG* in TT (*p* = 0.0464; HR = 0.60). Multivariable Cox proportional hazards model confirmed these findings for *CCAT1* in MT (*p* = 0.0005; HR = 0.002; CI (95%) = 0.00006–0.07) and detected associations between expression and DFS for *CCAT1* in MT (*p* = 0.0204; HR = 0.14; CI (95%) = 0.03–0.74) and *MIR155HG* in TT (*p* = 0.0366; HR = 0.18; CI (95%) = 0.04–0.90). Patients with higher expression of *MIR155HG* in TT were more likely to survive longer than those with lower *MIR155HG* expression. Yet, patients with higher *CCAT1* expression in MT had significantly shorter OS. These associations have been illustrated in Kaplan-Meier plots after applying an optimized threshold (Figure 4a,b). The resulting survival curves confirmed distinct differences in OS with regards to the expression of *CCAT1* in MT (*p* = 0.0017) and *MIR155HG* in TT (*p* = 0.0154).

**Figure 5:**
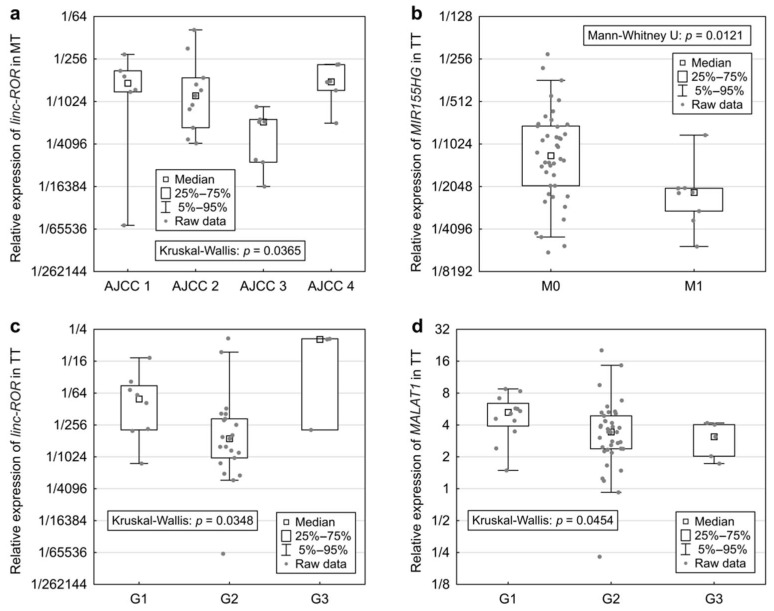
Associations of lncRNA expressions in MT and TT with tumour characteristics. Displayed are the relative expression values in the respective tissue type, grouped by different clinical characteristics. A higher value corresponds to higher expression. (**a**) The expression of *linc-ROR* in MT differed significantly within the categories of AJCC staging; (**b**) The expression of *MIR155HG* in TT was significantly higher in patients without distant metastases; (**c**) *linc-ROR* expression in TT also significantly differed between tumour grades; (**d**) *MALAT1* expression in TT was on the borderline of being associated with varying tumour grades.

For lncRNA expressions in MT, *linc-ROR* showed significant differences between the four AJCC stages (Kruskal-Wallis: *p* = 0.0365, Figure 5a). 

In TT, *MIR155HG* was significantly overexpressed in patients without metastases (M0) compared to those with metastases (Mann-Whitney U: *p* = 0.0121, Figure 5b). Expressions of *linc-ROR* and *MALAT1* in TT varied significantly with different tumour grade (Kruskal-Wallis: *p* = 0.0348 and *p* = 0.0454, Figure 5c,d). *Linc-ROR* was most expressed in G3 tumours and *MALAT1* expression, which was downregulated in tumour tissue, declined with tumour grade and reached the minimum in G3 tumours. Expression of *GAS5* differed among various localization of tumour and was higher in the right colon compared to left colon and rectum (Kruskal-Wallis: *p* = 0.0309).

### 2.4. Expression Ratios of Two lncRNAs in Non-Malignant and Tumour Tissues

To analyse the power of combining multiple lncRNAs, we used the ratio of two expression values and calculated the p-values for all possible combinations in MT and TT using a univariable Cox proportional hazard model. Due to the large-scale approach, the threshold for significance was adjusted to α = 0.01 (i.e., *p* < 0.01 was considered significant) but all *p*-values below 0.05 are noted. After testing all combinations of lncRNA ratios, we found no association (neither *p* < 0.01 nor *p* < 0.05) with DFS or OS in TT.

**Figure 6:**
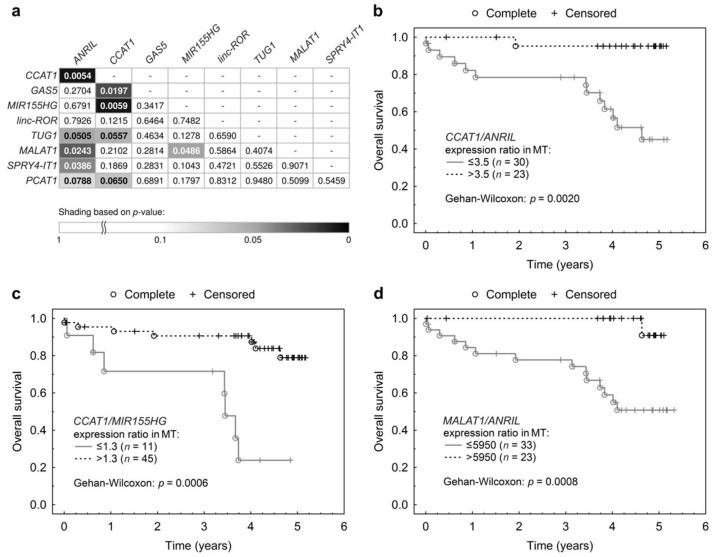
Associations of expression ratios of two lncRNAs in MT with OS. (**a**) Table displays all possible expression ratios of the nine lncRNAs with their respective univariable Cox proportional hazard model p-values for association with OS. Each cell contains the p-value of the expression ratio of lncRNAs stated in the corresponding row and column headings. The *p*-values are identical for reciprocal ratios, that is, for X/Y and Y/X; (**b**–**d**) Kaplan-Meier curves with an applied optimized threshold for the relationship between OS and the expression ratio of (**b**) *CCAT1*/*ANRIL* in MT, (**c**) *CCAT1*/*MIR155HG* in MT and (**d**) *MALAT1*/*ANRIL* in MT.

For lncRNA expression ratios in MT, two ratios were significantly associated with OS, *CCAT1*/*ANRIL* (*p* = 0.0054; HR = 0.65) and *CCAT1*/*MIR155HG* (*p* = 0.0059; HR = 0.68), with the latter being significant also in the multivariable Cox proportional hazards model (*p* = 0.0177; HR = 0.11, CI (95%) = 0.02–0.67). Four more ratios showed an indication of possible association with OS with *p* < 0.05 (Figure 6a): *CCAT1*/*GAS5* (*p* = 0.0197; HR = 0.67), *MALAT1*/*ANRIL* (*p* = 0.0243; HR = 0.64), *SPRY4-IT1*/*ANRIL* (*p* = 0.0386; HR = 0.67) and *MALAT1*/*MIR155HG* (*p* = 0.0486; HR = 0.65).

When an optimized threshold was applied for Kaplan-Meier curves, the most significant prognostic ratio for survival (*CCAT1*/*ANRIL*) showed a weak association with OS (Gehan-Wilcoxon: *p* = 0.0020, Figure 6b), whereas the ratios *CCAT1*/*MIR155HG* and *MALAT1*/*ANRIL* showed a stronger association with OS (Gehan-Wilcoxon: *p* = 0006 and *p* = 0.0008 respectively, Figure 6c,d). Additional Kaplan-Meier curves for lncRNA ratios can be found in Figure S2.

**Figure 7:**
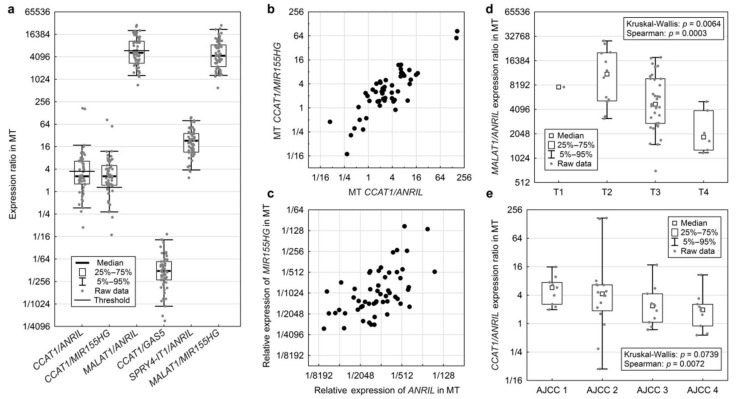
Expression ratios of two lncRNAs in MT. (**a**) Displayed are six lncRNA expression ratios in MT that are significantly or potentially associated with OS. For the ratios featured in Figure 6 the values of the optimized threshold used for the Kaplan-Meier curves are also shown; (**b**) Scatter plot for the correlation between the two most prognostic ratios in MT that are associated with OS; (**c**) Scatter plot of the two relative expression values of lncRNAs in MT involved in the two strongest ratios except *CCAT1*; (**d**) The ratio of *MALAT1*/*ANRIL* was negatively associated with tumour stage; (**e**) The expression ratio *CCAT1*/*ANRIL* significantly decreased with higher AJCC stage (Spearman: *p* = 0.0072).

All ratios of MT lncRNA expression that are associated with OS are displayed in Figure 7a. The two ratios with the highest association to OS show a strong mutual correlation (*CCAT1*/*MIR155HG* and *CCAT1*/*ANRIL*, *p* < 0.001, Figure 7b). This is due to two factors—first, the involvement of *CCAT1* in both ratios and second, the moderate correlation between the relative expressions of the two differing lncRNAs (Figure 7c). This indicates that both ratios might redundantly express the same prognostic feature. 

The six most prognostic expression ratios in MT were tested for correlations with clinical information. An association with T stage was detected for *MALAT1*/*ANRIL* (Kruskal-Wallis: *p* = 0.006, Figure 7d) with a decreasing trend with rising tumour stage (Spearman: *p* = 0.0003). 

Four expression ratios were associated with AJCC stage of the tumour: The strongest such association by Spearman correlation was exhibited by the ratio *CCAT1*/*ANRIL*, which decreased with rising AJCC stage (*p* = 0.0072, Figure 7e), followed by *CCAT1*/*MIR155HG* (*p* = 0.0218), *MALAT1*/*ANRIL* (*p* = 0.0239) and *SPRY4-IT1*/*ANRIL* (*p* = 0.0337). Only the strongest associations between expression ratios and clinical characteristics are displayed in Figure 7. Further data and figures for associations between lncRNA ratios in MT and clinical characteristics can be found in Figure S2.

No association was found for any of the tested ratios with G, N, M, age or tumour location.

## 3. Discussion

Advanced, metastatic CRC represents a lethal disease with 5-year survival rate about 12% and with severe limitations for therapeutical resection. Thus, the main goal of current CRC research comprises following priorities: (a) invention of new treatments and (b) invention of biomarkers for early diagnosis, discovery of new predictors that will allow us to efficiently use existing treatments and precise prognoses for the disease development. Given the emerging role of lncRNAs as important epigenetic regulators of tumour development and disease progression in multiple cancers, we aimed to explore the full potential of lncRNA expression and discovered several significant and potentially useful associations.

### 3.1. Differential Expression Values in TT Compared to MT

In our study, we confirmed that *CCAT1*, one of the best known lncRNAs in CRC, was upregulated in TT, similar to previously published results [16]. *CCAT1*, also known as *CARLo-5*, is a lncRNA of the human gene desert region 8q24.21, a region that contains enhancers and lncRNAs relevant to CRC and is known to influence cell growth and invasiveness and has shown relevance in CRC [16,20]. However, in our study its differential expression in TT was not associated with survival. 

The strongest association with survival was exhibited by the *MIR155HG* (*MIR155* host gene). This lncRNA is processed into the microRNA *miR-155* [23] which has been identified as a negative regulator of tumour protein 53 (TP53) and DNA mismatch repair (MMR) genes [24,25]. It is also known to be upregulated in many cancers and to promote migration and invasion of CRC cells [26]. However, recent studies have shown that *miR-155* may also work as a tumour suppressor. Kim et al. demonstrated that *miR-155* loss promotes tumour growth and Liu et al. found that overexpressed *miR-155* leads to apoptosis and suppresses cell proliferation [27,28]. Our comparison of *MIR155HG* expression in tissues showed a downregulation in TT, which was associated with shorter OS and DFS. Also, lower *MIR155HG* expression in TT was observed in patients with distant metastasis. This association probably resulted in the fact that *MIR155HG* expression change was not proved as independent predictor for neither OS nor DFS in multivariable Cox proportional hazards model. However, considering that the *MIR155HG* transcript is processed into *miR-155*, these findings support the observation that *miR-155* might work as a tumour suppressor. On the other hand, the high rate of processing of *MIR155HG* into *miR-155* might lead to a reduced number of full *MIR155HG* transcripts present in the tissue. In either case, the *MIR155HG* may be a promising biomarker to use in a longitudinal study with early stage patients, to investigate its potential to follow tumour progression and predict metastasis formation. 

Correlational analysis between *MIR155HG* fold change and other lncRNAs indicated a co-regulation with *ANRIL* in TT (*p* < 0.001, Spearman test). As stated above, the potential conversion from *MIR155HG* to *miR-155* has been identified as a driver for EMT in glioma [19]. *ANRIL* has been shown to influence the cadherin-switch in pancreatic cancer through inhibiting ATM-E2F1, thereby activating EMT [29]. So, this correlation might indicate the presence of EMT in the tumour and represent its metastatic potential. This is in line with results of two published articles in which *ANRIL* upregulation in CRC TT was associated with shorter survival and lymph node metastasis [17,30]. In our patients group, *ANRIL* also significantly varied with N stages of CRC patients but its differential expression decreased in patients with lymph node metastasis. Of note, though, our patient group had a different ethnical background and higher average age than those in the previously published CRC studies. *ANRIL* is also an epigenetic regulator of the tumour suppressor *CDKN2A/B* and therefore, influence cell proliferation [31]. However, Cunnington et al. observed that specific genetic variants may influence *ANRIL* expression and lower expression of *ANRIL* was associated with melanoma and cardiovascular disease [32]. 

*Linc-ROR* has been shown to act as both a repressor of p53 in CRC and a “sponge” for the tumour suppressor *miR-145*. *MiR-145* downregulates OCT4 and SOX2 through mRNA binding and degradation [13,18,33]. Our results confirm the upregulation of *linc-ROR* in tumour tissue of CRC patients across all stages. However, we observed higher *linc-ROR* expression in patients without distant metastases and lower AJCC stages. This observation is compromised by only six patients in the AJCC4/M1 group.

*PCAT1* was previously found to be upregulated in CRC and associated with poor survival [34] but in our patients we observed higher *PCAT1* expression associated with longer DFS. This observation contradicts presumed *PCAT1* function of repressing the tumour suppressor BRCA2 and serving as a sponge for microRNAs of the cell growth pathway [35,36] and shows the potential additional functions of *PCAT1* in CRC. 

In our study, *TUG1* expression did not vary in TT and was not associated with survival or other clinical characteristics except for age. However, existing studies report *TUG1* should be significantly upregulated in T and cell line experiments then documented that *TUG1* promotes cell proliferation and colon cancer cell migration [37,38].

*SPRY4-IT1* has been demonstrated to predict poor prognosis in CRC and promotes metastasis by enhancing cell proliferation, EMT gene expression and invasion [12,39]. Our differential expression values for *SPRY4-IT1* in TT show an upregulation but this difference was not significant in our samples. The association needs to be verified on a larger group of CRC patients.

LncRNA *GAS5* has been reported to have lower expression in TT and a negative correlation with OS. Cell line experiments have also pointed out that high *GAS5* expression reduces apoptosis and cell growth in CRC [40]. In our study, *GAS5* was equally expressed in MT and TT and did not reveal any association with survival or tumour stage.

*MALAT1* is known to be upregulated in many cancers and associated with poor survival and tumour growth [15,41]. In our study it was significantly, albeit minimally, downregulated in TT compared to MT and significantly upregulated in T4 tumours, suggesting its promotion of tumour growth. One limitation of our cohort is the limited group of progressed patients with metastases or large tumours, as the stated *MALAT1* studies were mainly composed of T4 stage patients with lymph node involvement [15]. Additionally, recent results have supported this theory by observing that *MALAT1* expression is lower in tissue samples of M0 or N0 stage but higher in cases with distant metastases in the lung or liver [42].

### 3.2. Normalized Expression in TT and MT

Second part of the study was focused on the individual tissue types and comparison of expression levels of target lncRNAs within either the non-malignant or the tumour tissue. In non-malignant tissue, although samples were macroscopically healthy, we should not exclude the possibility of micrometastatic infiltration. Information from non-malignant tissue can describe its fitness and may have an effect on patient´s prognosis. The normalized expression values showed only weak associations with survival and clinical characteristics. As stated above, differential expression of *CCAT1* was not associated with survival. However, increased expression of *CCAT1* in MT (but not in TT) was significantly associated with shorter OS in univariable and with both OS and DFS in multivariable models. To our knowledge, this type of analysis of normalized lncRNA expression in MT has not been previously considered and the unexpected result might open the door to a new approach for lncRNA expression assessment in carcinogenesis. Additionally, *linc-ROR* expression in MT was significantly different between the AJCC stages. While *linc-ROR* expression in TT increased from AJCC1 to AJCC3, expression decreased in MT between the stages. In TT, lower *MIR155HG* expression was weakly associated with shorter OS.

### 3.3. lncRNA Expression Ratios

Our results suggest that lncRNAs can be employed as biomarkers, especially in MT and that even those that had no significant association while comparing expression in tissues or by itself in MT, are now part of a strongly predictive ratio, like *CCAT1* or *ANRIL* in MT. For others, like *MIR155HG*, the association with survival was more pronounced by its use in an expression ratio in MT. The lncRNAs *MIR155HG*, *CCAT1* and *ANRIL* were found to be markers of early stage cancer development and were either responsible for promoting proliferation and migration (*CCAT1*, *ANRIL*) or observed to be associated with cancer in general (*MIR155HG*). 

## 4. Materials and Methods 

### 4.1. Patient Selection

In this retrospective study, we analysed 63 adult patients from the Czech Republic with pathologically confirmed colorectal cancer, operated between September 2012 and February 2014 at the University Hospital in Pilsen. Patients were stratified into all American Joint Committee on Cancer (AJCC) stages. All patients enrolled in the study agreed to the processing of their samples by signing informed consent. The study protocol was approved as a part of the project “Evaluation of molecular changes in tumour tissue and circulating tumour cells in prognosis and treatment optimization of colorectal cancer” by the ethics committee of the Faculty of Medicine and University Hospital in Pilsen on 12 August 2014 and complies with the International Ethical Guidelines for Biomedical Research Involving Human Subjects. Matching tissue samples from tumour and macroscopically non-malignant mucosa from the resected part of the colon (sampled as distant from the tumour as possible) were collected at the University Hospital in Pilsen during CRC resections. Anonymized clinical data were retrieved retrospectively from the hospital information system.

### 4.2. RNA Extraction

Frozen tissue samples of TT and MT were ground in liquid nitrogen and transferred into 1 mL of chilled TRI Reagent^®^RT (Molecular Research Centre, Inc., Cincinnati, OH, USA). RNA was then extracted following the manufacturer´s protocol and resuspended in molecular grade water. Isolated RNA was stored until further use in −80 °C freezer.

RNA concentration was measured with the Infinite M200 (Tecan Trading AG, Männedorf, Switzerland) set to the NanoQuant setting (260 nm absorbance) and purity was determined by the 230 nm/260 nm absorbance ratio. RNA integrity was tested by agarose gel electrophoresis. If degradation was detected, samples were excluded from further analysis.

### 4.3. RT-PCR

Reverse transcription PCR was performed using the RevertAid First Strand cDNA Synthesis Kit (ThermoFisher Scientific, #K1622, Waltham, MA, USA) and 500 ng of total RNA in a 20 μL reverse transcription reaction volume. Combined oligo (dT) and random hexamer primers were used, each with a final concentration of 2.5 μM, to prime the reverse transcription for both mRNAs and lncRNAs. Reverse transcription and quality control PCR were performed using the T100 PCR system (Bio-Rad Laboratories Inc., Hercules, CA, USA). Quality and purity (absence of DNA contamination) of the cDNA were measured by PCR using the PPP MasterMix (Top-Bio, s.r.o., Prague, Czech Republic) with GAPDH primers from the RevertAid First Strand cDNA Synthesis Kit (ThermoFisher Scientific Inc.). Control PCR was conducted for 40 cycles in 10 μL reactions using 5 μL of PPP MasterMix, 3.5 μL molecular water and 0.5 μL GAPDH primer mix with 1 μL of sample or reverse transcriptase negative control.

### 4.4. Quantitative-PCR (qPCR)

For the evaluation of lncRNA expression, gene-specific TaqMan^®^ Gene Expression probes from ThermoFisher (ThermoFisher Scientific Inc.) were used. Tested targets were *MIR155HG* (assay ID Hs01374569_ml), *TUG1* (Hs00215501_m1), *GAS5* (Hs03464472_m1), *ANRIL* (Hs04259476_m1), *MALAT1* (Hs01910177_s1), *SPRY4-IT1* (Hs03865501_s1) and *PCAT-1* (Hs04275836_s1). The probes for *CCAT1* and*linc-ROR* were purchased from GeneriBiotech (GENERI BIOTECH s.r.o, Hradec Králové, Czech Republic), order number 00491-14, assay ID hCCAT1_Q1 (reference sequence NR_108049.1, amplifying all transcript variants of *CCAT1*) and assay ID hLINC-ROR_Q2 (reference sequence NR_048536.1), respectively. For reference genes, previously tested *GAPDH* (assay ID Hs02758991_g1), *ACTB* (Hs01060665_g1) and *GUSB* (Hs00939627_m1) were used. Assays were tested for their reproducibility to assess optimal number of replicates for experimental settings.

Expression of lncRNAs was measured in duplicate (in 10 µL; 96 well plates) with the following reaction parameters: 2 min at 50 °C holding, 10 min at 95 °C holding, followed by 42 cycles of: 15 s at 95 °C and 1 min at 60 °C. We used the AppliedBiosystems^®^ 7500 Fast Real-Time PCR cycler (AppliedBiosystems Corp., Foster City, CA, USA) together with TaqMan^®^ Gene Expression Master Mix (ThermoFisher Scientific Inc.). All samples of cDNA were diluted 1:75 using 0.1 μg/mL yeast tRNA (ThermoFisher Scientific Inc.) in nuclease free water. Each reaction contained 4.5 μL of diluted sample, 5 μL of TaqMan^®^ Gene Expression Master Mix and 0.5 μL of the particular probe. Data analysis and manual quality control of automated thresholding was performed in the 7500 Software v1.5.1 (ThermoFisher Scientific Inc.).

### 4.5. Statistical Analysis

Duplicates of *C*_t_ values were averaged before further processing. *C*_t_ values of all three housekeeping genes were averaged and the result used as the final reference, that is, *C*_t_(ref). All subsequent analyses were performed with *C*_t_ values and their differences, in particular: (i) −∆∆*C*_t_ representing expression fold change (i.e., differential expression) in TT with respect to MT (−∆∆*C*_t_ = [*C*_tMT_(lncRNA) − *C*_tMT_(ref)] − [*C*_tTT_(lncRNA) − *C*_tTT_(ref)]); (ii) −∆*C*_t_ representing normalized expression in a single tissue type (−∆*C*_t_ = *C*_t_(ref) − *C*_t_ (lncRNA)); and (iii) negative value of the difference of lncRNA *C*_t_ values representing their ratio. The results of the analyses were converted into fold changes, normalized expressions and expression ratios as the last step before presentation while assuming 100% PCR efficiency (fold change = 2^−∆∆*C*t^; normalized expression = 2^−∆*C*t^; (expression A)/(expression B) = 2*^C^*^t(B)-*C*t(A)^).

Standard frequency tables and descriptive statistics were used to characterize the patient group. Significance of up- or downregulation of the lncRNAs in TT was assessed by testing the −∆∆*C*_t_ values against zero location with the Wilcoxon signed-rank test. Associations between expression descriptors and other clinical characteristics were analysed using the Mann-Whitney U test, Kruskal-Wallis test and Spearman’s correlation.

For the purpose of survival analysis, DFS was determined from the date of surgery to the date of disease recurrence or death. The date of recurrence was set to the average date between the last negative and the first positive examination if the interval between the examinations was 180 days or less. In cases of longer examination intervals, the recurrence date was set to 90 days before the first positive examination. OS was determined from the date of surgery to the date of death. Median follow-up was determined using the inverse Kaplan-Meier method. The significance of associations between lncRNA expression descriptors and survival times was assessed using a univariable Cox proportional hazards model. In order to visualize these associations with Kaplan-Meier survival estimation plots, a threshold value needed to be set for each prognostic variable to stratify the patients into two groups. This threshold was found by an automated optimization process implemented in Matlab (2014a, MathWorks Inc., Natick, MA, USA), in which the threshold value producing the smallest Log-rank *p*-value was determined and selected. Multivariable analysis of survival predictors was done using Cox proportional hazards model.

All reported *p*-values are two-tailed and the level of statistical significance was set to α = 0.05. Statistical analysis was performed in Statistica (version 12 Cz, TIBCO Software Inc., Palo Alto, CA, USA).

## 5. Conclusions

As emerging markers for various tumour types, lncRNAs are starting to be used alongside protein-coding genes for disease prognosis and prediction of treatment effect. In addition to the well-established differential expression between tumour and non-malignant tissue, the potential of lncRNAs in MT should be further studied, as it may give us information about the fitness of macroscopically unaffected collateral tissue and impact the patient prognosis after tumour removal.

The results of this study suggest that multi-variable analysis in a large scale lncRNA study might reveal various combinations of lncRNA up- or downregulation. The approach of using expression ratios instead of fold-change data also poses great advantages. First, it allows biomarker analysis by q-PCR without having to rely on the stability and validation of housekeeping genes expression which represents a common challenge and risk for misinterpretation in qPCR experiments [43]. Second, if two genes that have opposite prognostic effects are combined in an expression ratio, they will immensely enhance its predictive power, without the need for additional markers.

A prospective study on the roles of the *CCAT1*/*ANRIL* and *CCAT1*/*MIR155HG* ratios in non-malignant tissue in survival prediction should be conducted to replicate our results. The two main players, *MIR155HG* and *ANRIL*, should also be further analysed for their pathway interaction and influence of tumorigenesis. *PCAT1* and *MIR155HG* could even serve as predictive biomarkers for probable survival time and metastatic potential in CRC patients after tumour resection.

We admit that our patient group was rather limited, heterogeneous and the length of patient follow-up time was modest in relation to the OS and DFS times. Additionally, our cohort is ethnically homogeneous and results might not transfer to other ethnical groups. In future studies, we would also recommend including follow up sampling, blood sample analysis (as an easy accessible source of non-malignant cells) and deeper consideration of therapy.

## Figures and Tables

**Table 1:** Characteristics of the analysed colorectal cancer patients.

Characteristic	Category	Number	%
gender	F	24	38.1
	M	39	61.9
age (in years)	30–50	8	12.7
	50–70	32	50.8
	>70	23	36.5
T stage	T1	1	1.6
	T2	14	22.2
	T3	40	63.5
	T4	6	9.5
	unknown	2	3.2
N stage	N0	39	61.9
	N1	13	20.6
	N2	10	15.9
	unknown	1	1.6
M stage	0	47	74.6
	1	11	17.5
	unknown	5	7.9
tumour grade	G1	12	19.0
	G2	42	66.7
	G3	5	7.9
	unknown	4	6.3
AJCC staging	I	11	17.5
	II	19	30.2
	III	14	22.2
	IV	11	17.5
	unknown	8	12.7
primary tumour location	right or transversum	16	25.4
	Left or sigma	10	15.9
	rectum or rectosigma	31	49.2
	non-specific	6	9.5

## Supplementary Materials

Supplementary materials can be found at http://www.mdpi.com/1422-0067/19/9/2672/s1.

## Abbreviations

AJCC	American joint committee on cancer
ANRIL	CDKN2B antisense RNA 1
CCAT1	Colon cancer associated transcript 1
CCND	Cyclin D
CRC	Colorectal cancer
Ct	Cycle threshold
DFS	Disease free survival
EMT	Epithelial to mesenchymal transition
GAS5	Growth arrest-specific 5
HR	Hazard rate
Linc-ROR	Long intergenic non-protein coding regulator of reprogramming
lncRNA	Long non-coding RNA
MALAT1	Metastasis associated lung adenocarcinoma transcript 1
MIR155HG	MIR155 host gene
MMR	DNA mismatch repair
MT	Non-malignant (mucosa) tissue
OS	Overall survival
PCAT1	Prostate cancer associated transcript 1
q-PCR	Quantitative PCR
RT-PCR	Reverse transcription PCR
SPRY4-IT1	Sprouty homolog 4 intronic transcript 1
TP53	Tumour protein p53
TT	Tumour tissue
TUG1	Taurine up-regulated 1
