# Supplementary material for: lncRNAs in Non-Malignant Tissue Have Prognostic Value in Colorectal Cancer
Source: Int J Mol Sci. 2018 Sep 8;19(9):2672. doi: 10.3390/ijms19092672 (PMC6163783; doi:10.3390/ijms19092672)
Supplement: Supplementary file 1 [file ijms-19-02672-s001.pdf]

Table S1. List of all tested lncRNAs and their reported function in cancer.

lncRNA (abbr.)	lncRNA Full Name	Function	References
CCAT1	Colon Cancer Associated Transcript 1	Promoter of cell proliferation	[16]
Linc-ROR	Regulator of Reprogramming	Negative regulator of p53	[18]
MIR155HG	MIR155 Host Gene	Primary RNA of miR-155	[19]
TUG1	Taurine Up-Regulated 1	Promotes metastasis vie EMT	[11]
GAS5	Growth arrest specific 5	Cell proliferation, promotes metastasis	[10]
MALAT1	metastasis associated lung adenocarcinoma transcript 1	Promotes proliferation and metastasis; chemotherapy resistance and response marker	[15,20]
SPRY4-IT1	Sprouty homolog 4 intronic transcript 1	Promotes proliferation and invasion	[39]
PCAT1	Prostate Cancer Associated Transcript 1	Cell proliferation, apoptosis	[8]
ANRIL	antisense noncoding RNA in the INK4 locus	Cell migration and invasion (in vitro)	[17]

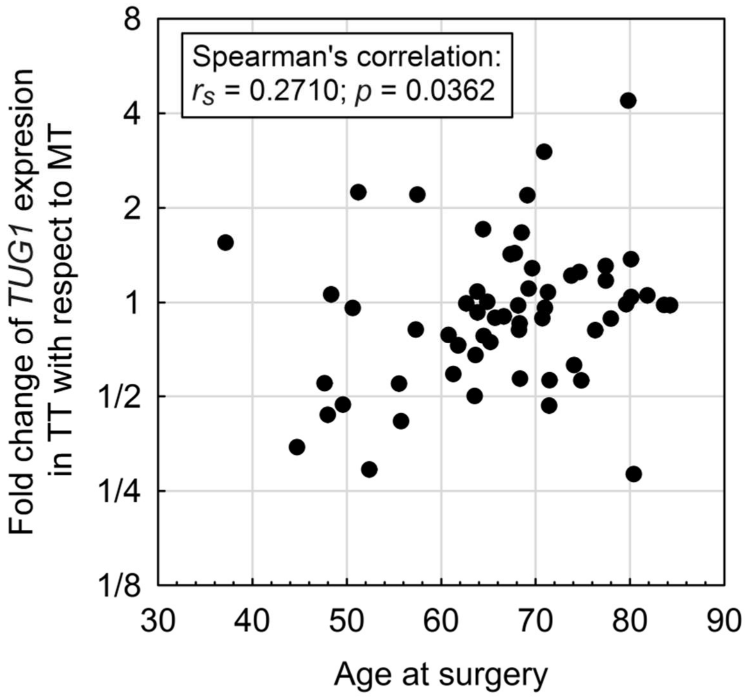

Figure S1. Correlation between differential expression of *TUG1* with age. Displayed is the association between patient age and differential expression of *TUG1*. With rising age, the fold-change of *TUG1* is more likely to rise as well.

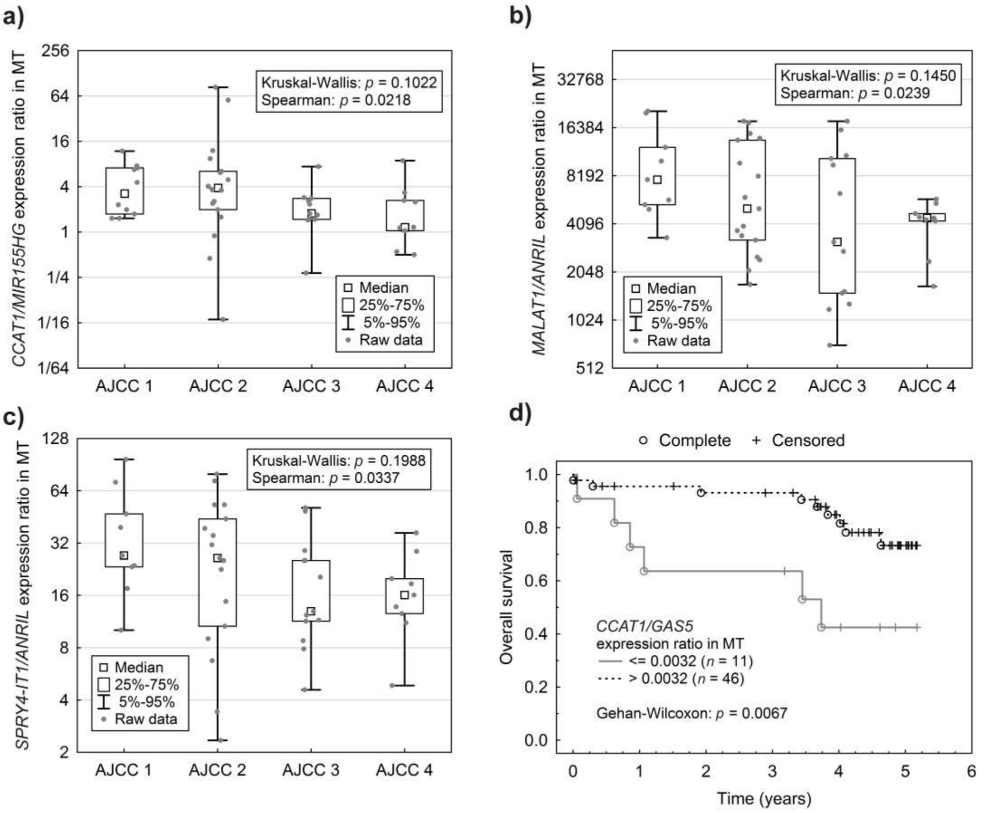

Figure S2. Association of lncRNA ratios with AJCC stage and OS. (**a**) Diagrammed is the association between the expression ratio of *CCAT1*/*MIR155HG* and AJCC stage. The ratio is likely to decline with rising AJCC stage; (**b**) The expression ratio of *MALAT1*/*ANRIL* is associated with AJCC stage and likely to decline with rising AJCC stage; (**c**) The expression ratio of *SPRY4-IT1*/*ANRIL* is associated to AJCC stage and is likely to be reduced in higher AJCC stages; (**d**) Optimized Kaplan-Meier curve for the expression ratio of *CCAT1*/*GAS5* showing its association with overall survival (OS).
